# Accuracy of a novel modified single computed tomography scanning method for assisting dental implant placement: a retrospective observational study

**DOI:** 10.1186/s40729-023-00509-8

**Published:** 2023-11-02

**Authors:** Hiroaki Shimizu, Takuya Mino, Yoko Kurosaki, Hikaru Arakawa, Kana Tokumoto, Aya Kimura-Ono, Kenji Maekawa, Takuo Kuboki

**Affiliations:** 1 Department of Oral Rehabilitation and Regenerative Medicine, Okayama University Graduate School of Medicine, Dentistry and Pharmaceutical Sciences, 2-5-1 Shikata-Cho, Okayama, 700-8525 Japan; 2 Department of Removable Prosthodontics and Occlusion, Osaka Dental University, 1-5-17 Otemae, Chuo-Ku, Osaka, 540-0008 Japan; 3 Center for Innovative Clinical Medicine, Okayama University Hospital, 2-5-1 Shikata-Cho, Okayama, 700-8525 Japan; 4 Department of Oral and Maxillofacial Surgery, Hyogo Medical University, 1-1 Mukogawa-Cho, Nishinomiya, 663-8501 Japan

**Keywords:** Dental implants, Implant placement, Accuracy, Radiographic guide, Surgical guide

## Abstract

**Purpose:**

The aim of this study is to compare dental implant placement accuracy of three surgical guide fabrication methods: single (SCT) and double computed tomography (DCT), and a newly developed modified SCT (MSCT) scan method.

**Methods:**

A total of 183 cases (183 surgical guides, and 485 implants) of static-guide-assisted implant placement surgery using the SCT, DCT, or MSCT methods in a dental clinic were included in the study. Three-dimensional (3D) deviations (mm) at the entry and tip of the implant body between preoperative simulation and actual placement were measured as surrogate endpoints of implant placement accuracy. The following survey details were collected from medical records and CT data: sex, age at implant placement surgery, surgical guide fabrication method, number of remaining teeth, implant length, implant location, alveolar bone quality, and bone surface inclination at implant placement site in preoperative simulation, etc. Risk factors for reducing implant placement accuracy were investigated using generalized estimating equations.

**Results:**

The SCT and DCT methods (odds ratios [ORs] vs. MSCT method: 1.438, 1.178, respectively), posterior location (OR: 1.114), bone surface buccolingual inclination (OR: 0.997), and age at implant placement surgery (OR: 0.995) were significant risk factors for larger 3D deviation at the entry; the SCT (OR: 1.361) and DCT methods (OR: 1.418), posterior location (OR: 1.190), implant length (OR: 1.051), and age at implant placement surgery (OR: 0.995) were significant risk factors for larger 3D deviation at the tip of the implant body.

**Conclusions:**

Implant placement accuracy was better using the MSCT method compared to the SCT and DCT methods.

## Background

In oral implant treatment, implant placement position has a significant effect on the safety of oral implant surgery and morphology of the final prosthesis. The determination of the three-dimensional (3D) bone morphology by a preoperative computed tomography (CT) scan and placement of the implant in an accurate position through preplanning with a simulation software program are needed for a successful oral implant surgery [1].

Currently, a static surgical guide, which is manufactured using computer-aided design/computer-aided manufacturing (CAD/CAM) technology based on a preoperative simulation, is widely used to achieve accurate implant placement. Existing static surgical guide fabrication methods are roughly classified into two types: a double computed tomography scan (DCT) method [2] and a single CT scan (SCT) method [3, 4].

The DCT method uses two types of CT images: a 3D CT image obtained with the patient wearing a template fabricated with polymerized acrylic resin and a 3D CT image of the template. In this method, the 3D digital imaging and communications in medicine (DICOM) data from the two images are simultaneously fused with the images of reference markers located in the template vestibule. Using this method, a surgical guide can be fabricated for any situation, from minor tooth loss to complete edentulism. However, the dimensional errors in surgical guides are not negligible in some clinical cases of the DCT method [5] because surgical guides were made from 3D CT images, wherein the threshold setting could affect the shape reproducibility. Block et al*.* also reported matching errors attributed to artifacts from the markers during CT scanning [6]. In addition, the presence of artifacts can conceal the 3D anatomical structures of soft and hard tissues, which may adversely affect diagnostic accuracy [3, 7].

The SCT method uses one type of CT image. To accurately reflect the preoperative simulation in the surgical guides, highly accurate overlapping 3D CT images (DICOM) and 3D oral plaster model (stereolithography; STL) surface images were obtained with reference to the surface morphology of the remaining teeth. This enabled the fabrication of a surgical guide from the intraoral surface STL data, which were unrelated to the CT-threshold settings. However, the SCT method is not applicable in complete edentulous patients and patients with a small number of remaining teeth owing to challenges in accurate data matching. Furthermore, metal artifacts appear frequently in the region with the remaining teeth on 3D CT images of patients with metal restorations, which reduces the matching point and matching accuracy.

Therefore, to solve all the aforementioned problems, a modified single CT (MSCT) scan method was developed and introduced [8]. The distinctive feature of the MSCT method is the use of a newly developed CT matching template (CTMT) with reference markers made of glass ceramics, which hardly generate artifacts [9]. In this method, three 3D images are superimposed: (1) a CT image (DICOM data) with the patient wearing a CTMT with glass ceramic reference markers, (2) a 3D surface image (STL data) of the patient’s oral plaster model without a CTMT, and (3) the STL data image with a CTMT. The matching accuracy can be improved using this superimposition method as the generated artifacts are extremely small. In addition, the artifacts during CT imaging in the simulation software program can be automatically deleted by a Boolean operation. The MSCT method has the advantages of both the SCT and DCT methods; in specific, it can be applied in all cases, from those involving minor tooth loss to complete edentulous (an advantage of the DCT method) patients, and it can be used to fabricate a surgical guide from high-resolution intraoral surface images (an advantage of the SCT method). However, the accuracy of guided surgery using the MSCT method has not been evaluated in comparison with that of other existing surgical guide fabrication methods.

Therefore, we conducted a retrospective observational study to evaluate the accuracy of computer-guided implant surgery using a surgical guide fabricated by the MSCT method in comparison with using the DCT and SCT methods.

## Methods

### Cases

The survey included all the cases who underwent static-guide-assisted oral implant placement surgery at Shimizu Dental Clinic (one facility in Japan) from March 1, 2014, to March 1, 2018, using one of the following CT scanning methods: (1) SCT method, (2) DCT method, or (3) MSCT method. One surgeon well-trained for static surgical guides (H.S.) decided the most suitable computer-guided system for each case according to the patient’s oral condition before the surgery. From March 2014 to September 2015, the DCT method was only used. In October 2015, the SCT and MSCT methods were introduced. Thereafter, an appropriate method was chosen from the DCT, SCT, and MSCT methods. The SCT method was only applied to cases with one or more untreated natural teeth (far from artifacts) in each of the 3 portions, anterior teeth, and right and left molars. From December 2016, the SCT and MSCT methods were applied except for the DCT method because the MSCT method gained credibility. The exclusion criteria were as follows: (1) cases in whom the guided surgery system could not automatically match the DICOM data between the pre- and post-operative CT scans and (2) those who did not provide their consent to participate in the study.

The research protocol was approved by the Okayama University Ethics Committee (Ethics Committee No. 14000046, Approval Number: 1806-031). Patients provided written informed consents with permission to use their data for scientific purposes.

### Treatment steps

Treatment steps for the SCT method were as follows. A preoperative CT scan (Aquilion Lightning, Canon Medical Systems, Japan) was performed to obtain the DICOM data of maxillofacial region. Definitive impressions were taken using silicone impression materials (Imprint^TM^4 Penta™ Soft Tray, 3M EPSE, Aquasil Ultra, Dentsply Sirona, USA) to fabricate plaster cast model of intraoral morphology. The obtained plaster cast model was scanned with 3D desktop scanner (CARES^®^ Scanner D7 plus, Straumann, Switzerland), then converted to STL data. The DICOM data of the maxillofacial region and STL data of the plaster cast model were imported to a simulation software program (coDiagnostiX^®^, Dental Wings Inc., Canada) and superimposed with reference to the surface morphology of the remaining teeth. The implant placement simulation and surgical guide design were performed based on the superimposed data. Surgical guide was designed to fit to the STL data of the intraoral morphology and fabricated using a 3D printer (CARES^®^ P Series P40, Straumann, Switzerland).

In the DCT method, definitive impression was taken using silicone impression materials (Imprint^TM^4 Penta™ Soft Tray, 3 M EPSE, Aquasil Ultra, Dentsply Sirona, USA) to fabricate the intraoral plaster cast model. A dental technician fabricated a radiographic guide, by burying 6–8 gutta-percha on the plaster cast model. Two types of CT scan, with a radiographic guide and the patient wearing the radiographic guide, were performed (Aquilion Lightning, Canon Medical Systems, Japan), and their DICOM data were obtained. Two types of DICOM data were imported to the simulation software program (Nobel Clinician^®^, Nobel Biocare, Switzerland) and superimposed with reference to the gutta-percha points. After performing the implant placement simulation and surgical guide design, the surgical guide was fabricated based on the DICOM data of the radiographic guide using the optical shaping method (Nobel Biocare, Switzerland).

The MSCT method was performed according to the protocol previously described by Shimizu et al. [8]. Definitive impression was taken using silicone impression materials (Imprint^TM^4 Penta™ Soft Tray, 3 M EPSE, Aquasil Ultra, Dentsply Sirona, USA) to fabricate the plaster cast model of the intraoral morphology. The obtained plaster cast model was scanned using a 3D desktop scanner (CARES^®^ Scanner D7 plus Straumann, Switzerland) and converted to STL data. Resin template for the CT scan was printed using a 3D printer (Form 2, Formlabs, USA) based on the obtained STL data of the intraoral morphology. Six glass ceramics markers were added on the occlusal surface of the resin template. Finally, CTMT was completed. CT scan (Aquilion Lightning, CANON MEDICAL SYSTEMS, Japan) of the patient wearing CTMT was performed. Two types of scanning, plaster cast model itself and CTMT attached plaster cast model, were performed (CARES^®^ Scanner D7 plus, Straumann, Switzerland). Then, the obtained STL and DICOM data of the patient wearing the CTMT were imported to the simulation software program (coDiagnostiX^®^, Dental Wings Inc., Canada) and superimposed with reference to the glass ceramics markers and remaining teeth morphology. After implant placement simulation, the surgical guide design was performed based on the STL data of the intraoral morphology, and the surgical guide was fabricated by a 3D printer (CARES^®^ P Series P40, Straumann, Switzerland).

The surgical guides were fabricated using each methodology, and their internal fittings were confirmed through the pre-formed inspection windows before surgery. During oral implant surgery, all the surgical guides were anchored using fixation pins. The implant surgeries were open-flap or flapless, according to the recommended guided surgery protocol of the manufacturer. Implant bodies were placed without the removal of the surgical guide. A postoperative CT scan was taken to confirm whether the actual implant position was clinically appropriate. Placed implants were selected from following systems: Brånemark System, NobelActive, NobelSpeedy, NobelReplace Straight, NobelReplace/Select Tapered, NobelReplace Conical Connection (Nobel Biocare, Switzerland), and Straumann Bone Level and Bone Level Tapered (Straumann, Switzerland).

### Complications

The following events described in the medical records were considered as complications. (1) complications before surgery: (a) failure of the preoperative simulation for designing surgical guides due to artifacts, (2) complications during implant placement surgery: (a) unexpected changes in the implant placement surgery plans (unplanned bone augmentations or changes in implant body diameter or width) during the surgery, (b) fracture of surgical guide templates during the implant surgery (c) unexpected implant body exposure from the alveolar bone surface, (d) perforation into the maxillary sinus or nasal cavity during implant placement surgery, and (e) collision of the implant body with an adjacent tooth.

### Observation factors

**Fig. 1:**
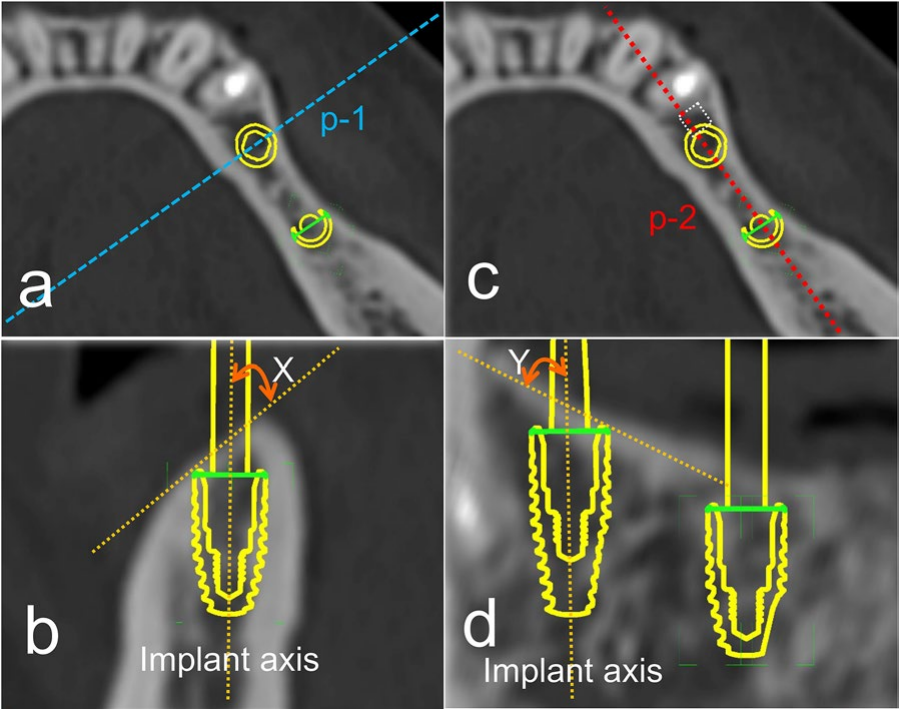
Measurement method of alveolar bone surface inclination. **a** A buccolingual virtual plane (p-1) was set through the implant body axis in the axial slice at the entry of the implant body in the preoperative simulation. **b** Buccolingual bone surface inclination X (0–90°) was measured between the implant axis and bone surface line on p-1. **c** A mesiodistal virtual plane (p-2) was set through the implant body axis in the axial slice at the entry of the implant body in the preoperative simulation. **d** Mesiodistal bone surface inclination Y (0–90°) was measured between the implant axis and bone surface line on p-2

Alveolar bone quality according to Lekholm and Zarb (type 1/2/3/4) [10] and alveolar bone surface inclination (degree) was evaluated from a preoperative CT image. Bone surface inclination at the implant placement site in preoperative simulation was evaluated separately as buccolingual (bone surface inclination X) and mesiodistal inclination (bone surface inclination Y) (Fig. 1).

Other information obtained from an electronic medical database for regular implant treatments with reference to the previous reports [11] was as follows: patient’s age at implant placement surgery; sex; surgical guide fabrication method (SCT/DCT/MSCT); number of remaining teeth, coronal teeth (number of remaining teeth with coronal structure and dummy teeth in fixed partial dentures), and teeth with metal restorations (including zirconia restorations); number of placed implants; dentition defect type (Kennedy classification Class I/II/III/IV/complete edentulism); number of fixation pins used during the implant placement surgery; shape of the implant body (straight/tapered); implant width (narrow platform [NP]/regular platform [RP]/wide platform [WP]); implant length; implant location (anterior/posterior, maxillary/mandibular); whether or not immediate implant placement was performed; whether or not bone augmentation was performed; and distance between guide sleeve bottom to bone surface (4.0/5.5/6.0 mm).

### Preoperative planning and measurement of 3D deviation between preoperative simulation and actual placement of the implant body (surrogate endpoints)

**Fig. 2:**
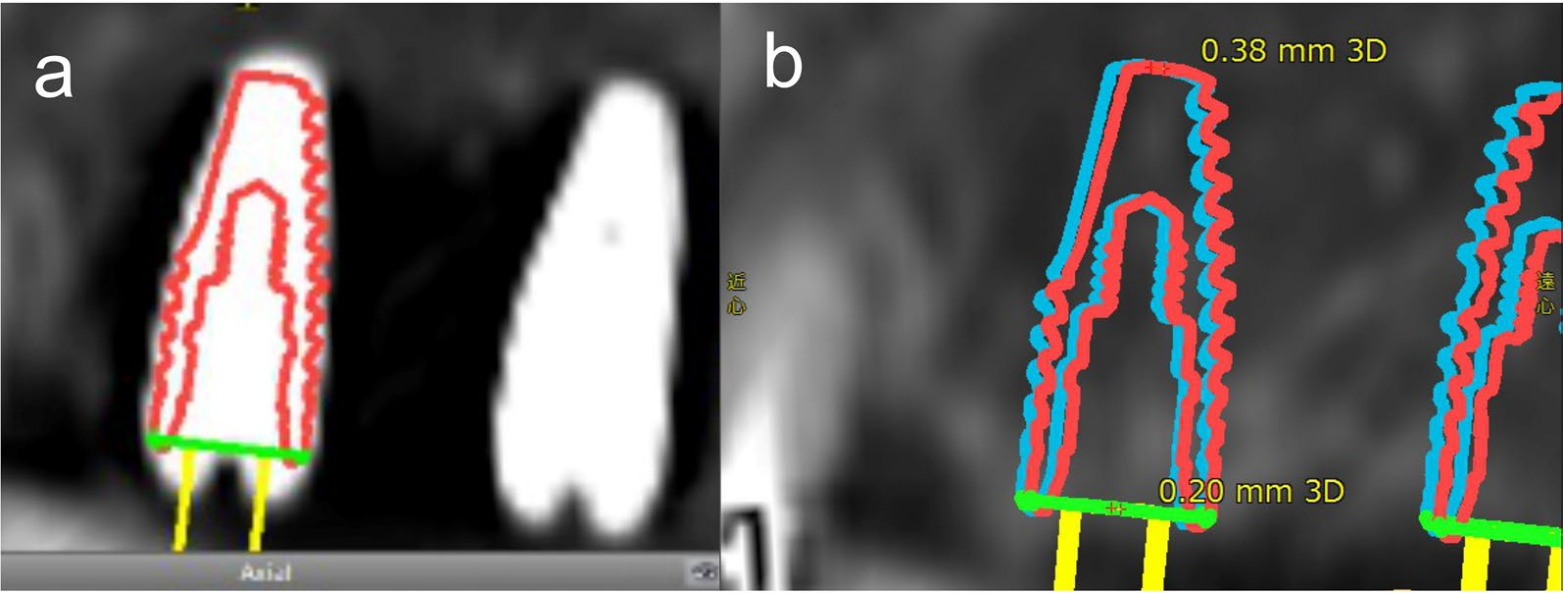
Measurement method of the 3D deviation between preoperative simulation and placed implant body position with coDiagnostiX^Ⓡ^. **a** A pseudo-implant body was placed based on the actual implant shadow on the CT image after implant placement. **b** Automatic calculated 3D deviation at the entry and tip between the planned implant body and actual implant body placement position. (Red: Placed implant body; Blue: Planned implant body). 3D, three-dimensional; CT, computed tomography

The planning software program, coDiagnostiX^Ⓡ^, was used to measure the 3D deviations (mm) at the entry and tip of the implant body between the preoperative simulation and actual placement position by the SCT and MSCT methods. The measurement protocol described by Monaco et al*.* (2020) was applied to this study [12]. The DICOM data of the pre- and postoperative CT images were assessed using coDiagnostiX^Ⓡ^, which were adjusted to the same CT threshold and then automatically and accurately superimposed with reference to the characteristic anatomical morphology on CT images using a software function. The pseudo-implant body was accurately placed on the actually placed implant body on the postoperative CT images (Fig. 2a). The 3D positional deviations and distances between the preoperative simulation and actual placement position of the implant bodies were measured automatically using the “Treatment Evaluation” function. The X (mesiodistal axis), Y (buccolingual axis), and Z deviations (depth axis) were automatically measured at the position of entry and tip of the planned and placed implant bodies, and the 3D deviation (3D = $\sqrt{{\mathrm{X}}^{2}+{\mathrm{Y}}^{2}+{\mathrm{Z}}^{2}}$) was automatically calculated (Fig. 2b).

**Fig. 3:**
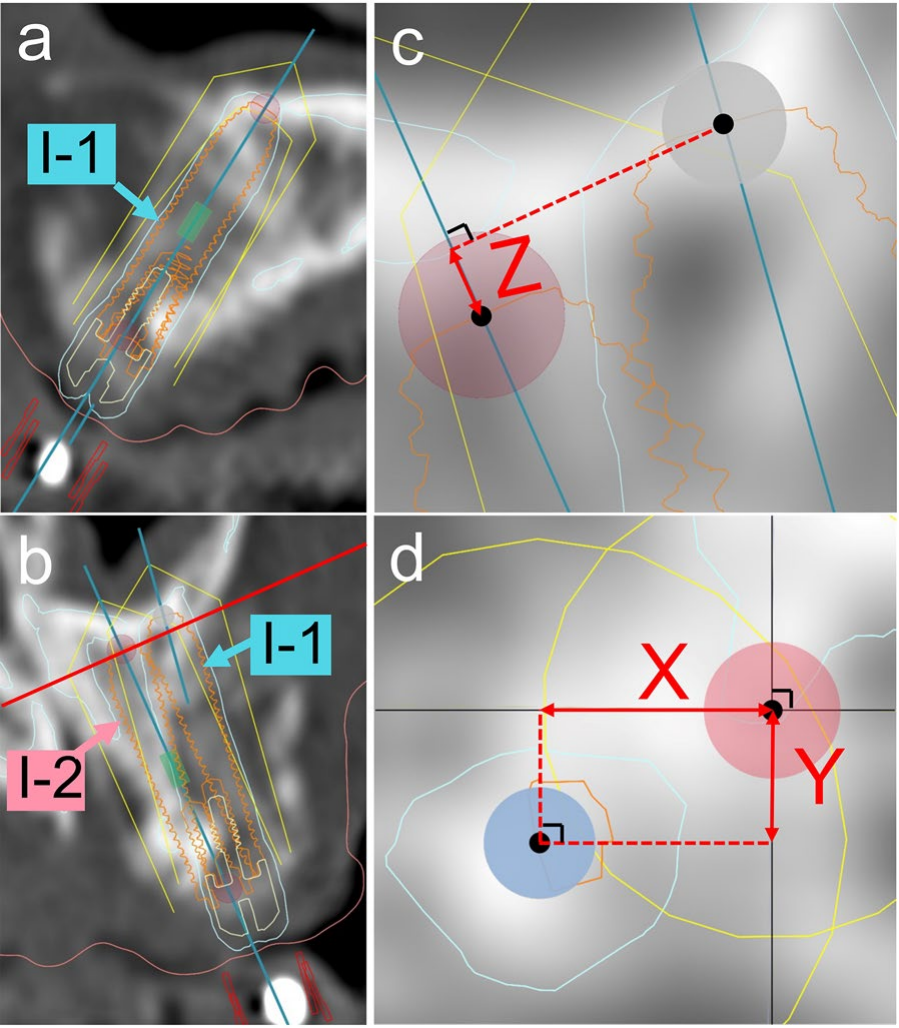
Measurement method of 3D deviation between preoperative simulation and placed implant body position with Nobel Clinician^Ⓡ^. **a** A pseudo-implant body (I-1) was placed based on the actual implant shadow on the CT image after implant placement. **b** A postoperative pseudo-implant body (I-1) on the cross-sectional slice through the planned implant body (I-2) axis. **c** Vertices of implant bodies of **b** were enlarged. 2D deviation of the depth (Z) at the tip between the preoperative and postoperative pseudo-implant body was manually measured using the distance measurement tool. **d** The axial slice which was contacted to tip of planned implant body (I-2) and rectangular to planned implant (I-2) axis, red line of **b**. The mesiodistal 2D deviation (X) and buccolingual 2D deviation (Y) at the tip between the preoperative and postoperative pseudo-implant body were manually measured using the distance measurement tool. 3D, three-dimensional; 2D, two-dimensional; CT, computed tomography

In the DCT method, the planning software program, Nobel Clinician^Ⓡ^, was used to measure the 3D deviations at the entry and tip of the implant body between the preoperative simulation and actual placement position. The measurement protocol described by Verhamme et al. (2015) was applied to this study [13]. Superimposition of the pre- and postoperative DICOM data and pseudo-implant body placement were performed using the same method as that used for coDiagnostiX^Ⓡ^ (Fig. 3a). In contrast to the method used with coDiagnostiX^Ⓡ^, the 3D positional deviations and distances between the actually placed implant body and preoperative simulation were measured manually. The X, Y, and Z deviations at the position of entry and tip between the planned and placed implant bodies were measured with a distance measuring tool, and the 3D deviation was calculated manually using the same formula (Fig. 3b–d).

Measurements of the 3D deviations between the preoperative simulation and actual placement position of the implant bodies were performed by two examiners (T. M. and Y. K.) independently, while they were not informed about the applied surgical guide fabrication methods. The average of the deviation values measured by the two examiners was adopted as the 3D deviation of each implant.

### Statistical analysis

The Kruskal–Wallis and chi-square tests were used to compare the observation factors among the SCT, DCT, and MSCT methods. Since there were no missing data among all observation factors of patients, statistical correction was not needed.

The inter-rater reliability in the 3D deviation measurements of the planned and placed implant bodies between the two examiners was assessed using the Intraclass correlation coefficient (ICC) for each planning software program.

The Steel–Dwass test was used to compare the median 3D deviation between the planned and placed implant bodies among the SCT, DCT, and MSCT methods. Kruskal–Wallis test, Mann–Whitney U test, or Spearman’s rank correlation coefficient was used to analyze the relationships between the predictor variables and the 3D deviations between the planned and placed implant positions at the entry and tip of the implant body between each surgical guide fabrication method.

To evaluate the accuracy of implant placement position using each surgical guide fabrication method, generalized estimating equations (GEEs) were used to identify the risk factors for large 3D deviations at the entry and tip of the implant body between the preoperative simulation and actually placed position. GEEs were implemented using the forced entry method.

Statistical analyses were performed using SPSS for Windows (version 25 for SPSS, IBM, Japan). Steel–Dwass test was performed using (JMP version 11, SAS, Japan). The level of significance was set at *p* < 0.05.

## Results

### Cases and complications

A total of 183 cases (158 patients) met the selection criteria and underwent implant surgeries (average age at implant placement surgery: 62.3 ± 11.5 years, male/female: 54/104, 183 surgical guides, and 485 implants).

Of the 183 surgical guides, five DCT surgical guides, involving nine implant bodies, induced complications. The complications were as follows: (1) inability to fabricate the surgical guide due to artifacts (one surgical guide, one implant), (2) collision with adjacent teeth due to displacement of the implant drilling position during the guided surgery (three surgical guides, four implants), and (3) application of bone augmentation for unexpected bone defects due to drilling errors (one surgical guide, four implants). Of the three cases (five surgical guides) with complications, cases (1) and (3) were excluded from the analyses as the pre- and postoperative CT data could not be superimposed.

The number of analyzed cases was 181 (156 patients, average age at implant placement surgery: 61.7 ± 11.8 years, male/female: 54/102, 181 surgical guides, and 480 implants). Table 1 shows the basic data for each surgical guide fabrication method. Significant differences were observed between the surgical guide fabrication methods, except for in terms of sex, age at implant placement surgery, number of teeth with metal restorations, implant location (anterior/posterior), and presence or absence bone augmentation (Table 1).

**Table 1:** Basic data of each surgical guide fabrication method

Factors related to each surgical guide	SCT method	DCT method	MSCT method	*p* value
	*n* = 27	*n* = 88	*n* = 66	
Sex (male/female; number of subjects)	12/15	31/57	21/45	0.541^*^
Age at implant placement surgery (mean ± SD in years)	55.1 ± 13.2	63.6 ± 10.2	63.3 ± 11.5	0.022^†^
Number of remaining teeth (mean ± SD)	11.4 ± 1.6	8.0 ± 3.9	7.7 ± 4.3	**< 0.001**^**†**^
Number of coronal teeth (mean ± SD)	11.6 ± 1.7	8.5 ± 4.0	8.7 ± 4.5	**< 0.001**^**†**^
Number of teeth with metal restorations (mean ± SD)	2.3 ± 2.2	4.4 ± 3.3	3.9 ± 3.3	0.014^†^
Number of placed implants (mean ± SD)	2.0 ± 1.2	3.3 ± 2.1	2.7 ± 1.9	**< 0.001**^**†**^
Dentition defect type (Kennedy Classification Class I/II/III/IV/complete edentulism; number of subjects)	3/6/16/2/0	14/28/23/12/11	11/20/21/3/11	< 0.001^*^
Number of fixation pins (mean ± SD)	2.9 ± 0.9	5.8 ± 2.9	5.3 ± 2.8	**< 0.001**^**†**^
Factors related to each implant body	SCT method	DCT method	MSCT method	*p*-value
	*n* = 44	*n* = 268	*n* = 168	
Shape of implant body (straight/tapered; number of subjects)	0/44	62/206	0/168	< 0.001^*^
Implant width (NP/RP/WP; number of subjects)	10/32/2	75/157/36	64/101/3	< 0.001^*^
Implant length (mean ± SD)	10.4 ± 1.3	12.4 ± 2.2	10.9 ± 1.9	**< 0.001**^**†**^
Implant location (anterior/posterior; number of subjects)	10/34	81/187	41/127	0.312^*^
Implant location (maxillary/mandibular; number of subjects)	19/25	163/105	79/89	0.006^*^
Immediate implant placement (with/without; number of subjects)	0/44	23/245	4/164	0.001^*^
Bone augmentation (with/without; number of subjects)	5/39	16/252	14/154	0.382^*^
Alveolar bone quality (1/2/3/4; number of subjects)	1/5/34/4	12/76/90/90	2/33/111/22	< 0.001^*^
Distance between guide sleeve bottom to bone surface (4.0/5.5/6.0 mm; number of subjects)	18/0/26	0/268/0	87/0/81	< 0.001^*^
Bone surface inclination X (0 ≤ X ≤ 90 degree; mean ± SD)	64.0 ± 17.5	70.2 ± 17.1	63.2 ± 17.2	**< 0.001**^**†**^
Bone surface inclination Y (0 ≤ Y ≤ 90 degree; mean ± SD)	69.4 ± 12.6	75.5 ± 14.4	72.3 ± 14.1	**< 0.001**^**†**^

Bold means *p* < 0.05

CT: computed tomography, SCT: single CT scan, DCT: double CT scan, MSCT: modified single CT scan, NP: narrow platform, RP: regular platform, WP: wide platform, SD: standard deviation

^†^Kruskal–Wallis test

^*^Chi-square test

### Comparison of 3D deviation between planned and placed implant position (univariate analysis)

The 3D deviations between the planned and placed implant positions at the entry and tip of the implant body between each surgical guide fabrication method were compared using the Steel–Dwass test. As a result, the median 3D deviations of the SCT method at the entry was 0.788 mm (first quartile: 0.623, third quartile: 1.008) and that at the tip was 1.084 mm (first quartile: 0.742, third quartile: 1.269). The median 3D deviation of the DCT method at the entry was 0.646 mm (first quartile: 0.428, third quartile: 0.891) and that at the tip was 1.100 mm (first quartile: 0.727, third quartile: 1.433). The median 3D deviations of the MSCT method at the entry was 0.522 mm (first quartile: 0.338, third quartile: 0.738) and that at the tip was 0.674 mm (first quartile: 0.451, third quartile: 0.991). The results showed that the median 3D deviations of the MSCT method (entry: 0.522, tip: 0.674) was significantly smaller than that of the SCT (entry: 0.788, tip: 1.084) and DCT (entry: 0.646, tip: 1.100) methods at both the entry and tip of the implant body (Table 2, *p* < 0.01).

**Table 2:** Comparison of 3D deviation by each surgical guide fabrication method

		The entry of implant body										The tip of implant body									
		*n*	Mean ± SD	Quantiles					95% CI	*p* value		*n*	Mean ± SD	Quantiles					95% CI	*p* value	
				0%	25%	Median	75%	100%						0%	25%	Median	75%	100%			
a	SCT method	44	0.821 ± 0.431	0.108	0.623	0.788	1.008	2.315	0.690–0.952	a–b	0.059	44	0.996 ± 0.440	0.033	0.742	1.084	1.269	2.215	0.863–1.130	a-b	0.430
										a–c	**< 0.001**									a-c	**< 0.001**
b	DCT method	268	0.690 ± 0.399	0.000	0.428	0.646	0.891	2.259	0.643–0.739	b–a	0.059	268	1.131 ± 0.545	0.050	0.727	1.100	1.433	3.496	1.066–1.198	b-a	0.430
										b–c	**0.002**									b-c	**< 0.001**
c	MSCT method	168	0.564 ± 0.304	0.077	0.338	0.522	0.738	1.978	0.518–0.610	c–a	**< 0.001**	168	0.742 ± 0.394	0.091	0.451	0.674	0.991	2.091	0.682–0.803	c-a	**< 0.001**
										c–b	**0.002**									c-b	**< 0.001**

Bold means *p* < 0.05

3D: three-dimensional, CT: computed tomography, SCT: single CT scan, DCT: double CT scan, MSCT: modified single CT scan, SD: standard deviation, CI: confidence interval

Steel–Dwass test

The ICC for inter-examiner reliability was 0.949 for coDiagnostiX^Ⓡ^ and 0.951 for Nobel Clinician^Ⓡ^. According to Landis and Koch, both reliability levels can be estimated as “almost perfect” [14].

### Examination of factors affecting implant accuracy (multivariate analysis with GEEs)

The following predictor variables were submitted initially as the factors related to each surgical guide: surgical guide fabrication method (SCT/DCT/MSCT), sex (male/female), age at implant placement surgery, number of coronal teeth, number of teeth with metal restorations, number of placed implants, dentition defect type (Kennedy Classification Class I/II/III/IV/complete edentulism), and number of fixation pins used during surgery; further, the following were submitted as the factors related to each implant body: implant body shape (straight/tapered), width (NP/RP/WP), length, and location (anterior/posterior, maxillary/mandibular); immediate implant placement (with/without); bone augmentation (with/without); alveolar bone quality; distance between the guide sleeve bottom to bone surface; and bone surface inclination (X, Y).

The relationships between the predictor variables and the 3D deviations between the planned and placed implant positions at the entry and tip of the implant body among each surgical guide fabrication methods are shown in Table 3.

**Table 3:** The relationships between predictor variables and the 3D deviations between the planned and placed implant positions at the entry and tip of the implant body among each surgical guide fabrication methods

	Large 3D deviation at the entry of the implant body			Large 3D deviation at the tip of the implant body		
	Mean ± SD	Correlation coefficient	*p* value	Mean ± SD	Correlation coefficient	*p* value
Factors related to each surgical guide						
Sex						
Male	0.642 ± 0.386	–	0.325^‡^	0.976 ± 0.514	–	0.763^‡^
Female	0.667 ± 0.377	–		0.987 ± 0.524	–	
Age at implant placement surgery	–	− 0.102	**0.025**^**§**^	–	− 0.082	0.073^§^
Number of coronal teeth	–	− 0.137	**0.003**^**§**^	–	− 0.074	0.106^§^
Number of teeth with metal restorations	–	− 0.159	**< 0.001**^**§**^	–	− 0.037	0.418^§^
Number of placed implants	–	0.094	**0.039**^**§**^	–	0.056	0.224^§^
Dentition defect type						
Class I	0.572 ± 0.335	–	**0.002**^**†**^	0.883 ± 0.443	–	**0.006**^**†**^
Class II	0.680 ± 0.399	–		0.996 ± 0.524	–	
Class III	0.586 ± 0.333	–		0.890 ± 0.447	–	
Class IV	0.691 ± 0.428	–		1.217 ± 0.622	–	
Edentulism	0.761 ± 0.389	–		1.044 ± 0.559	–	
Number of fixation pins	–	0.137	**0.003**^**§**^	–	0.141	**0.002**^**§**^
Factors related to each implant body						
Shape of implant body						
Straight	0.665 ± 0.405	–	0.876^‡^	1.167 ± 0.642	–	**0.028**^**‡**^
Tapered	0.657 ± 0.376	–		0.956 ± 0.495	–	
Implant width						
NP	0.676 ± 0.376		0.702^†^	1.003 ± 0.522	–	0.836^†^
RP	0.649 ± 0.383	–		0.972 ± 0.514	–	
WP	0.660 ± 0.376	–		0.993 ± 0.567	–	
Implant length	–	0.103	**0.024**^**§**^	–	0.253	**< 0.001**^**§**^
Implant location						
Anterior	0.682 ± 0.370	–	0.301^‡^	1.018 ± 0.545	–	0.472^‡^
Posterior	0.649 ± 0.383	–		0.970 ± 0.510	–	
Implant location						
Maxillary	0.678 ± 0.383	–	0.203^‡^	1.031 ± 0.525	–	**0.021**^**‡**^
Mandibular	0.635 ± 0.375	–		0.926 ± 0.510	–	
Immediate implant placement						
With	0.833 ± 0.395	–	**0.011**^**‡**^	1.288 ± 0.503	–	**0.002**^**‡**^
Without	0.648 ± 0.377	–		0.965 ± 0.516	–	
Bone augmentation						
With	0.657 ± 0.405	–	0.81^‡^	1.095 ± 0.720	–	0.510^‡^
Without	0.658 ± 0.378	–		0.974 ± 0.501	–	
Alveolar bone quality	–	− 0.008	0.859^§^	–	− 0.009	0.839^§^
Distance between guide sleeve bottom to bone surface	–	0.070	0.123^§^	–	0.051	0.262^§^
Bone surface inclination X	–	− 0.109	**0.017**^**§**^	–	− 0.020	0.664^§^
Bone surface inclination Y	–	− 0.141	**0.002**^**§**^	–	− 0.087	0.057^§^

Bold means *p* < 0.05

3D: three-dimensional, NP: narrow platform, RP: regular platform, WP: wide platform

^†^Kruskal–Wallis test

^‡^Mann–Whitney U test

^§^Spearman’s rank correlation coefficient

Table 4 shows the results of the GEE analysis for the risk factors for large 3D deviations at the entry of the implant body between the preoperative simulation and actual placement position. The SCT (*p* < 0.001) and DCT (*p* = 0.006) methods were compared to the MSCT method; posterior implant position (*p* = 0.034), smaller bone surface inclination X (*p* = 0.006), and younger age at implant placement surgery (*p* = 0.036) were significant risk factors for large 3D deviations.

**Table 4:** Risk factors for large 3D deviation at the entry of the implant body between preoperative simulation and actual placement analyzed by GEEs

	*p* value	Odds ratio	95% CI	
			Lower limit	Upper limit
Factors related to each surgical guide				
Surgical guide fabrication methods (SCT method/DCT method/MSCT method)				
SCT method	**< 0.001**	1.438	1.177	1.757
DCT method	**0.006**	1.178	1.047	1.326
MSCT method		1.000		
Sex (male/female: male)	0.529	0.969	0.880	1.068
Age at implant placement surgery (younger)	**0.036**	0.995	0.991	1.000
Number of coronal teeth (lower)	0.131	0.981	0.957	1.006
Number of teeth with metal restorations (higher)	0.892	1.001	0.983	1.020
Number of placed implants (lower)	0.602	0.990	0.955	1.027
Dentition defect type (Kennedy classification Class I/II/III/IV/complete edetulism)				
Class I	0.404	0.924	0.766	1.113
Class II	0.598	1.071	0.830	1.381
Class III	0.830	0.968	0.720	1.301
Class IV	0.835	1.034	0.756	1.414
Edentulism		1.000		
Number of fixation pins (higher)	0.258	1.019	0.986	1.052
Factors related to each implant body				
Shape of implant body (straight/tapered: straight)	0.793	0.982	0.855	1.128
Implant width (NP/RP/WP)				
NP	0.746	1.026	0.877	1.201
RP	0.835	0.985	0.858	1.132
WP		1.000		
Implant length (longer)	0.938	0.999	0.976	1.022
Implant location (anterior/posterior: posterior)	**0.034**	1.114	1.007	1.206
Implant location (maxillary/mandibular: maxillary)	0.726	0.974	0.838	1.131
Immediate implant placement (with/without: with)	0.292	0.898	0.734	1.098
Bone augmentation (with/without: with)	0.961	1.005	0.819	1.233
Alveolar bone quality (lower)	0.785	1.009	0.947	1.075
Distance between guide sleeve bottom to bone surface (longer)	0.922	1.003	0.942	1.068
Bone surface inclination X (smaller)	**0.006**	0.997	0.994	0.999
Bone surface inclination Y (smaller)	0.397	0.999	0.997	1.001

Bold means *p* < 0.05

3D: three-dimensional, GEEs: generalized estimating equations, CI: confidence interval, CT: computed tomography, SCT: single CT scan, DCT: double CT scan, MSCT: modified single CT scan, NP: narrow platform, RP: regular platform, WP: wide platform

Table 5 shows the results of the GEE analysis for the risk factors for 3D deviations of the tip of the implant body. The SCT method (*p* = 0.003) and DCT method (*p* < 0.001) were compared to MSCT method, posterior implant location (*p* = 0.001), longer length of the implant body (*p* = 0.006), and younger age at implant placement surgery (*p* = 0.043) were significant risk factors for large 3D deviations.

**Table 5:** Risk factors for large 3D deviation at the tip of the implant body between preoperative simulation and actual placement analyzed by GEEs

	*p* value	Odds ratio	95% CI	
			Lower limit	Upper limit
Factors related to each surgical guide				
Surgical guide fabrication methods (SCT method/DCT method/MSCT method)				
SCT method	**0.003**	1.361	1.109	1.670
DCT method	**< 0.001**	1.418	1.230	1.633
MSCT method		1.000		
Sex (male/female: male)	0.770	0.982	0.870	1.109
Age at implant placement surgery (younger)	**0.043**	0.995	0.989	1.000
Number of coronal teeth (lower)	0.981	1.000	0.969	1.032
Number of teeth with metal restorations (higher)	0.899	0.998	0.974	1.023
Number of placed implants (lower)	0.222	0.977	0.942	1.014
Dentition defect type (Kennedy Classifications Class I/II/III/IV/complete edentulism)				
Class I	1.193	0.856	0.677	1.082
Class II	0.934	1.013	0.754	1.360
Class III	0.538	0.902	0.650	1.252
Class IV	0.492	1.107	0.829	1.477
Edentulism		1.000		
Number of fixation pins (higher)	0.113	1.035	0.992	1.079
Factors related to each implant body				
Shape of implant body (straight/tapered: straight)	0.710	1.035	0.862	1.243
Implant width (NP/RP/WP)				
NP	0.433	1.096	0.871	1.380
RP	0.669	1.046	0.851	1.286
WP		1.000		
Implant length (longer)	**0.006**	1.051	1.014	1.088
Implant location (anterior/posterior: posterior)	**0.001**	1.190	1.074	1.319
Implant location (maxillary/mandibular: maxillary)	0.240	0.886	0.725	1.084
Immediate implant placement (with/without: with)	0.488	0.917	0.718	1.171
Bone augmentation (with/without: with)	0.464	0.904	0.690	1.184
Alveolar bone quality (lower)	0.976	1.001	0.917	1.093
Distance between guide sleeve bottom to bone surface (longer)	0.906	1.005	0.930	1.086
Bone surface inclination X (smaller)	0.419	0.999	0.996	1.002
Bone surface inclination Y (smaller)	0.088	0.998	0.995	1.000

Bold means *p* < 0.05

3D: three-dimensional, GEEs: generalized estimating equations, CI: confidence interval, CT: computed tomography, SCT: single CT scan, DCT: double CT scan, MSCT: modified single CT scan, NP: narrow platform, RP: regular platform, WP: wide platform

## Discussion

This retrospective observational study aimed to evaluate the differences in the accuracy of the implant placement position between the newly developed MSCT method and existing surgical guide fabrication methods. Most of the previous studies, which evaluated the effect of the surgical guide system on implant placement accuracy, focused on comparisons between accuracy with and without a surgical guide [15]. None of the studies compared the placement accuracies between different surgical guide fabrication methods. Therefore, this is the first study to include the types of static surgical guide fabrication methods as explanatory variables and to identify the risk factors that might reduce implant placement accuracy. The details of each issue investigated in this study are discussed below.

### Comparison of 3D deviation between planned and placed implant positions (univariate analysis)

Inter-examiner reliability in evaluating the 3D deviation between the preoperative simulation and actual implant placement positions was assessed. The ICC of both methods, using coDiagnostiX^Ⓡ^ and Nobel Clinician^Ⓡ^, was more than 0.9, which indicates that both measurement methods are sufficiently reliable.

Regarding the DCT method, Van Assche et al. (2012) reported that the mean 3D deviation at the entry of the implant body was 0.99 mm and that at the tip of the implant body was 1.24 mm [16]. According to a meta-analysis of 3D deviations at the implant body placement position on using the DCT and SCT methods, the mean 3D deviations were 1.2 and 1.4 mm at the entry and tip, respectively [17]. In this study, the mean 3D deviations of the SCT method was 0.82 mm at the entry and 1.00 mm at the tip and that of the DCT method was 0.69 mm at the entry and 1.13 mm at the tip. Taking these into consideration, implant placement surgeries with the SCT and DCT methods in this study produced equivalent or higher accuracies than those previously reported. Nevertheless, the MSCT method showed significantly smaller 3D deviations than the SCT and DCT methods at both the entry and tip of the implant body. These results indicate the effectiveness of the MSCT method. However, since this study was an observational study, it must be noted that the baseline demographic data of patients in each study group based on the applied surgical guide were different. Since there were significant differences in the distribution of the baseline data between the three groups, the difference in 3D deviations between them might not have originated from the accuracy of the surgical guide fabrication method. Future studies that address this drawback are necessary.

### Factors related to implant placement accuracy (multivariate analysis with GEEs)

The predictors employed in this study were factors related to both the surgical guides and implant bodies. Thus, we utilized GEEs in this study, which can be used to analyze the effects of both categories of related factors separately, instead of a multiple regression analysis. We considered that employing this statistical analysis method would be substantially advantageous because the mean number of implants placed per surgical guide was 2.9 ± 1.9, implying that one surgical guide was prepared for the placement of multiple implant bodies in this study.

According to the results of GEEs, the application of the MSCT method can significantly reduce the 3D deviations between pre- and postoperative implant positions considering the surgical guide fabrication methods. A possible reason for the reduction in the 3D deviation was the difference in the imaging matching procedure employed in the MSCT method. As mentioned above, we utilized newly developed reference markers made of glass ceramics, which reduce the production of artifacts in the matching process between the 3D CT image and 3D intraoral surface shape image in the MSCT method. By contrast, both the images were superimposed based on the 3D images of the remaining teeth in the SCT method. However, with this procedure, matching errors may occur owing to the artifacts generated from metal restorations on these teeth [18]. In cases involving the DCT method, artifacts generated from the markers for image matching may induce matching errors. In addition, the fact that surgical guides can be produced by the morphology built from 3D CT image to be influenced by CT-threshold settings can be considered to be related to implant placement accuracy in these cases.

Derksen et al. (2019) reported that 3D deviations at both the entry and tip of the implant body significantly increased in cases with 5–6 unrestored remaining teeth compared to in those with 7 or more unrestored remaining teeth when using the SCT method [11]. In addition, Nabha et al. (2014) demonstrated that the greater the number of restored remaining teeth, the greater the number of generated metal artifacts; this causes matching errors between the 3D CT image and 3D surface image of the remaining teeth (STL) [19]. However, the numbers of remaining teeth with coronal structures and metal restorations were not identified as the significant risk factors for large 3D deviations in this study. A possible reason for this phenomenon was that this study included patients who underwent procedures using the MSCT and DCT methods, which are not affected by the artifacts caused by metal restorations during the matching process.

Implant placement in the posterior location was a significant risk factor for increased 3D deviation at the entry and tip of the implant body. When placing implants in the posterior region, confirming whether the hand drill is positioned correctly to fit the guide sleeve against the anterior region could be more challenging. In addition, drill entry angles are more susceptible to restriction due to the shorter distance between the alveolar crest at the placement site and opposing teeth. Thus, 3D deviations might increase when implants are placed in the posterior region. Furthermore, placement of a longer implant body was a significant risk factor for increased 3D deviation at the tip of the implant body. When drilling is angled, the 3D deviations at the tip between the preoperative simulation and postoperative implant placement sites could increase with longer implant placement. This finding is consistent with that in a study by D’haese et al. (2012), which utilized the NobelGuide system [20].

Larger alveolar bone surface inclination at the placement site was a significant risk factor for an increase in 3D deviations at the entry of the implant body. A previous report suggested a certain degree of variable range between a guide sleeve and drill, which can cause an error between preoperatively planned and actual implant placement sites [21]. Moreover, a mechanical engineering report by Sakuma et al. (1983) demonstrated that a drill hole was formed at a deviation from the target position during drilling on an inclined surface [22]. Once the drill hole was deviated, they concluded that the amount of cumulative displacement could not be corrected. In addition, Ohnishi et al. (2003) reported that a disproportion of the radial force at the starting point of drill penetration occurred on inclined surfaces, thereby decreasing the drilling accuracy [23]. Hence, the steep inclination of the alveolar bone surface might cause drilling and implant placement errors owing to the sliding of the drill apex and implant body from the target position, causing subsequent changes in direction. Further studies are required to confirm these findings. Further, age at implant placement surgery was identified as a risk factor for an increase in 3D deviations at the tip and the entry of the implant body. This had no multicollinearity with other observational factors; thus, it was completely independent. Previous reports showed that cancellous bone tissue structure became sparse with aging [24] and cancellous bone became low density and more porous [25]. Thus, younger people’s hard bone tissue structure and their high bone density might affect to these results.

Suspected observation factors, which could affect implant placement accuracy, were not identified as significant risk factors, e.g., coronal teeth, dentition defect type, number of fixation pins, and distance between sleeve bottom to bone surface according to the GEE analyses. However, it should not be interpreted that these factors never affect implant placement accuracy. According to the basic data, significant correlations or differences were observed between the surgical guide fabrication methods and these suspected observation factors. Considering these relationships, the GEE analyses might have been affected by multicollinearity. Thus, the surgical guide fabrication method, which was the most affected factor among mutually related observation factors, was only identified as a significant risk factor for implant placement accuracy.

### Complications

In this study, severe complications, such as a collision of the placed implant body with an adjacent tooth, unexpected changes in the implant surgery plan, and unexpected bone augmentation procedures due to drilling errors during surgeries, were seen only in the cases where the DCT method was applied. In this study, the median values of the 3D deviation in the DCT group were almost identical to those in the SCT group. However, the range from minimum to maximum 3D deviations in the DCT group was wider than that in the SCT group. In addition, the DCT method was selectively applied to cases with large edentulous spaces in this study. In these cases of large edentulous areas, when the surgical guide is placed in the oral cavity, it is generally difficult to confirm whether it is precisely restored to the planned position. Therefore, the occurrence of severe complications was observed in the DCT group.

### Limitations

In this study, two examiners measured the 3D deviations between the preoperatively planned and actual implant positions in each case, while not being informed about the surgical guide fabrication method. However, the examiners could predict the implant system and type of surgical guide in the cases where the DCT method was applied because Nobel Clinician^Ⓡ^, the planning software program, was only used for the measurement of DCT method. Two type of software programs, coDiagnostiX^Ⓡ^ and Nobel Clinician^Ⓡ^, were used to measure the outcomes of this study. Both the methodologies were utilized in previously reported outcome [12, 13, 26, 27] measurements but the degree of coincidence between the two has not been evaluated. Even though this research was a retrospective study, there were no missing data because the clinic that conducted this study had regularly constructed the clinical database for implant treatment. This enabled compensation of the shortcomings of retrospective studies. However, since every surgery was performed by one operator (H.S.), there was a limitation in generalizing the findings of this study.

## Conclusions

This retrospective observational study demonstrated that the MSCT method significantly improves the implant placement accuracy at both the entry and tip of the implant body, compared with the DCT and SCT methods. Severe complications were observed only in the cases where the DCT method was applied.

## Data Availability

The datasets generated and analyzed during the current study are available from the corresponding author on reasonable request.

## Abbreviations

3D: Three-dimensional
SCT: Single computed tomography
DCT: Double computed tomography
MSCT: Modified single computed tomography
DICOM: Digital imaging and communications in medicine
STL: Stereolithography
CTMT: Computed tomography matching template
NP: Narrow platform
RP: Regular platform
WP: Wide platform
ICC: Intraclass correlation coefficient
GEE: Generalized estimating equation
OR: Odds ratio
